# Surveillance and laboratory collaboration in response to an outbreak of *Vibrio parahaemolyticus*, *Plesiomonas shigelloides*, and *Aeromonas hydrophila* in Sekondi-Takoradi, Ghana: a case series

**DOI:** 10.1186/s13256-021-03243-0

**Published:** 2022-01-27

**Authors:** Michael Owusu, Bernard Nkrumah, Ebenezer Kofi Mensah, Jones Lamptey, Godfred Acheampong, David Sambian, Augustina Sylverken, Shannon Emery, Lucy Maryogo Robinson, Solomon Asante Sefa, Eric Amoako, Irene Amedzro, Slyvester Chinbuah, Kwame Asante, Yaw Adu-Sarkodie, David Opare

**Affiliations:** 1 Centre for Health System Strengthening, Kumasi, Ghana; 2 African Field Epidemiology Network, Accra, Ghana; 3 Sekondi Public Health Laboratory, Ghana Health Service, Sekondi, Ghana; 4 Association of Public Health Laboratories, Silver Spring, MD USA; 5 Department of Clinical Microbiology, Kwame Nkrumah University of Science and Technology, Kumasi, Ghana; 6 National Public Health and Reference Laboratory, Ghana Health Service, Accra, Ghana; 7 Department of Medical Diagnostics, Kwame Nkrumah University of Science and Technology, Kumasi, Ghana; 8 Department of Theoretical and Applied Biology, Kwame Nkrumah University of Science and Technology, Kumasi, Ghana

**Keywords:** *Vibrio parahaemolyticus*, *Plesiomonas shigelloides*, *Aeromonas hydrophila*, Public Health Laboratory, Case Report

## Abstract

**Background:**

The detection of epidemic-prone pathogens is important in strengthening global health security. Effective public health laboratories are critical for reliable, accurate, and timely testing results in outbreak situations. Ghana received funding as one of the high-risk non-Ebola affected countries to build and strengthen public health infrastructure to meet International Health Regulation core capacities. A key objective was to build laboratory capacities to detect epidemic-prone diseases.

**Case presentation:**

In June 2018, a local hospital received eight patients who presented with acute diarrhea. A sample referral system for Ghana has not been established, but the Sekondi Zonal Public Health Laboratory staff and mentors collaborated with Disease Surveillance Officers (DSOs) to collect, package, and transport stool specimens from the outbreak hospital to the Public Health Laboratory for laboratory testing. The patients included seven females and one male, of Fante ethnicity from the Fijai township of Sekondi-Takoradi Municipality. The median age of the patients was 20 years (interquartile range: 20–29 years). *Vibrio parahaemolyticus* was identified within 48 hours from four patients, *Plesiomonas shigelloides* from one patient, and *Aeromonas hydrophila* from another patient. There was no bacteria growth from the samples from the two other patients. All patients were successfully treated and discharged.

**Conclusion:**

This is the first time these isolates have been identified at the Sekondi Zonal Public Health Laboratory, demonstrating how rapid response, specimen transportation, laboratory resourcing, and public health coordination are important in building capacity towards achieving health security. This capacity building was part of the United States Centers for Disease Control and Prevention engagement of international and local partners to support public health laboratories with supplies, diagnostic equipment, reagents, and logistics.

## Introduction

Following the 2003 severe acute respiratory syndrome (SARS) outbreak, the World Health Organization (WHO) drafted the International Health Regulations 2005 (IHR), requiring member countries to prevent, rapidly detect, respond, and report outbreaks and public health emergencies [1, 2]. Ghana received funding from the Global Health Security Agenda (GHSA) to support strengthening of IHR capacities and, in line with the WHO Regional Office for Africa (WHO/AFRO), adopted the Integrated Disease Surveillance and Response (IDSR), with an aim to strengthen indicator-based surveillance and build a comprehensive public health response system [3].

One of the key pillars of IDSR is to have a strong and efficient public health laboratory that has the capacity to identify, confirm, and report priority pathogens to the appropriate units.

Efficient laboratories are critical for reliable, accurate, and timely testing of suspected outbreak cases. Ghana has a network of laboratories comprising both clinical laboratories within health care facilities and separate Public Health Laboratories (PHL) at the zonal level.

However, observations show that the role of the PHLs in supporting epidemiologic surveillance of disease in Ghana faces challenges, including lack of mechanisms to transport samples to the laboratory, inappropriate sample collection, lack of logistics to support surveillance, limited financial support, and weak communication between laboratory personnel and disease surveillance officers [4].

As part of strengthening the laboratory and epidemiologic surveillance of priority pathogens in Ghana, the United States Centers for Disease Control and Prevention (US CDC) engaged international [Association of Public Health Laboratories (APHL)] and local [Centre for Health System Strengthening (CfHSS)] partners to build the capacity of PHLs to detect epidemic-prone infectious diseases in Ghana.

We report here the detection of *Vibrio parahaemolyticus*, *Plesiomonas shigelloides*, and *Aeromonas hydrophila* through a concerted effort of disease surveillance officers (DSOs), PHL staff, and laboratory mentors.

## Case presentation

**Fig. 1:**
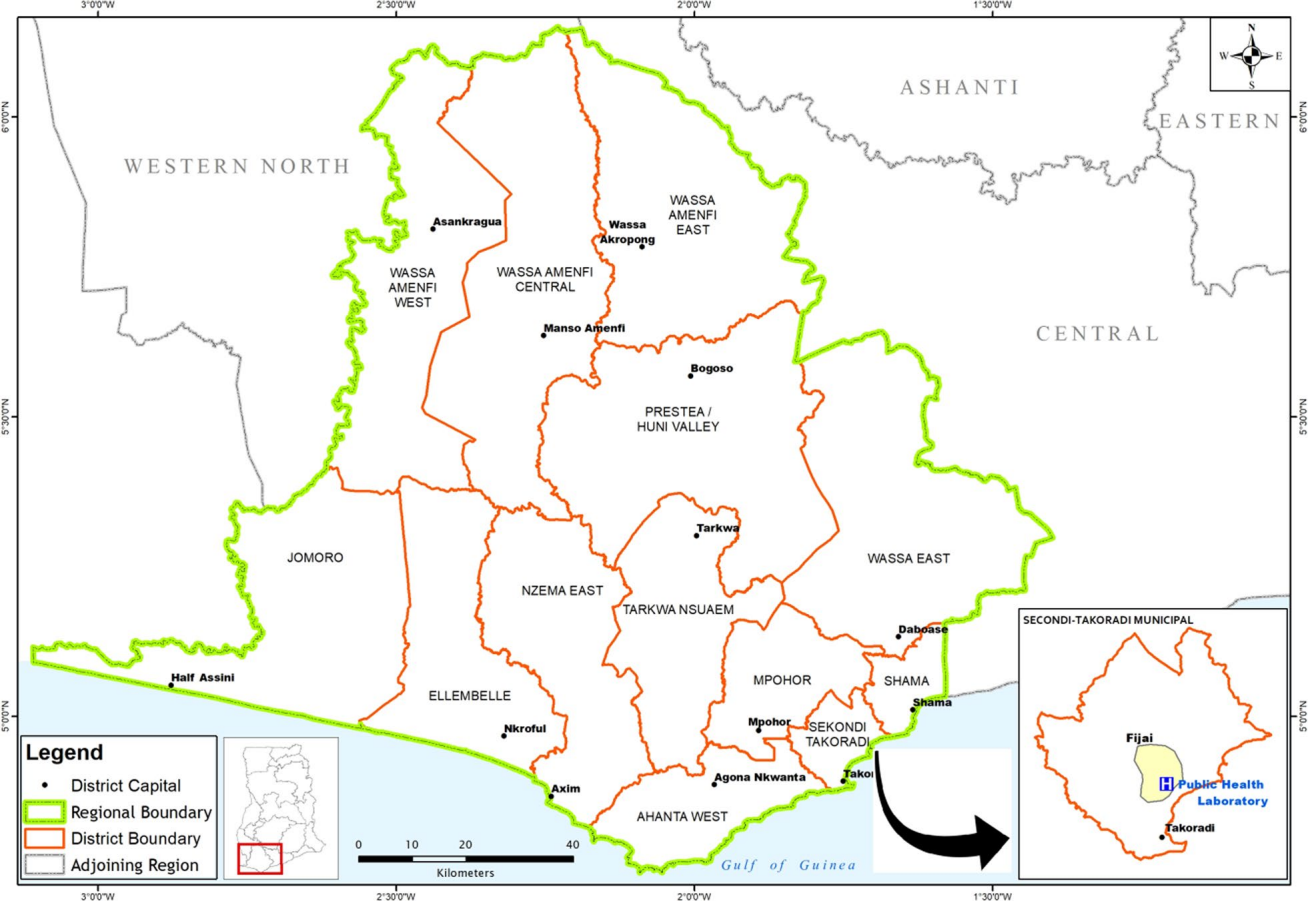
Map showing Fijai Community within the Sekondi-Takoradi Municipality. (This map is an original work generated by the study team)

On the 29 June 2018, the Holy Child Hospital (a private health facility) in the Sekondi-Takoradi Municipality of the Western Region received eight acutely-ill patients who presented with general weakness and diarrhea. On direct questioning, it was reveled that all the patients belonged to the Fante ethnic group, and came from the Fijai vicinity of the Sekondi-Takoradi Municipality (Fig. 1). Two patients ate *kenkey* (a local meal made from maize) with fish from the same vendor, one ate food prepared at home, and the rest ate *waakye* (local meal made with rice and red beans) with fish from another food vendor. All the food vendors were from the Fijai community in the municipality. On examination, all patients appeared weak and had watery, mucoid diarrhea. None of the patients had fever (axillary temperature of 38 °C and above, by WHO’s definition), and their blood pressure readings were within normal range. The hospital notified the DSOs about the suspected outbreak cases, who in turn called the laboratory staff to assist with the investigation. Five stool samples and three rectal swabs from all eight patients were collected, labelled, and transported (at 4 °C) to the Sekondi Zonal PHL in Cary–Blair transport medium. It took approximately 30 minutes for the samples to arrive at the testing laboratory. All the samples were accompanied by case report forms.

The samples were immediately cultured on xylose lysine deoxycholate agar (XLD) and thiosulfate citrate bile salt sucrose agar (TCBS) and incubated at 37 °C overnight. Mixed colonies (yellow and pink) were seen on the XLD plates and pure cultures were observed on the TCBS plates. The pinkish colonies on XLD were subcultured onto fresh XLD media and incubated overnight at 37 °C to produce pure colonies. All isolates that grew on TCBS appeared tiny and green depicting possible nonsucrose fermentation. Isolates on XLD showed tiny, pink appearance with no hydrogen sulfide (H_2_S) production. Single well-isolated colonies from each XLD and TCBS plate were subcultured onto separate blood agar plates and incubated overnight at 37 °C. Poly O1 *Vibrio* grouping was performed on all the isolates for detection of *Vibrio cholerae,* but all were negative. Colonies of bacteria were inoculated in triple sugar iron (TSI) agar, citrate, urease media, and sulfur indole motility (SIM) medium. Oxidase test was further conducted on the isolates. Antimicrobial susceptibility Ttest (AST) was performed for ceftazidime (30 µg), ciprofloxacin (5 µg), meropenem (10 µg), gentamicin (10 µg), cefotaxime (30 µg), and tetracycline (30 µg) using the Kirby–Bauer disc diffusion method on Mueller Hinton agar. Interpretation of the ASTs was done following guidelines from the Clinical and Laboratory Standards Institute (CLSI) [5]. *Escherichia coli* ATCC 25922 was used as the quality control organism for the antimicrobial susceptibility test.

Seven of the patients were females and one was male. Their median age was 20 years (interquartile range: 20–29 years). The bacteria isolates appeared as Gram negative rods with all isolates showing glucose fermentation, oxidase positive, and motility positive. Results from citrate test and urease reaction were all negative. Triple sugar iron (TSI) presentation of all the isolates revealed an alkaline-over-acid (K/A) reaction, with no gas released and no H_2_S produced. Using the analytical profile index (API) 20NE (Biomerieux, France) system, the isolates were identified as *V. parahaemolyticus*, *P. shigelloides*, and *A. hydrophila*. *V. parahaemolyticus* was the most predominant (4/8; 50%), followed by both *P. shigelloides* (1/8; 12.5%) and *A. hydrophila* (1/8; 12.5%). Two (2/8; 25%) samples did not yield any enteropathogen. All three pathogens were sensitive to all antimicrobial agents, except for tetracycline, which was not effective against *V. parahaemolyticus*, or *A. hydrophila*.

Patients whose samples yielded enteropathogens were treated with oral and intravenous medications such as ciprofloxacin, metronidazole, oral rehydrated salts (ORS) with zinc, omeprazole, and metoclopramide. The two individuals with no enteropathogens in their specimens were put on only fluid replacement therapy (ORS + zinc) and were discharged after diarrhea had ceased.

## Discussion

Ghana received funding as one of the high-risk non-Ebola affected countries, to build and strengthen public health infrastructures to meet IHR core capacities. A key objective was to build laboratory capacities to detect epidemic-prone diseases. To achieve this, the US CDC engaged APHL, which in turn contracted a Ghana in-country partner CfHSS to support the PHLs with supplies, diagnostic equipment, reagents, and logistics. CfHSS recruited mentors and supported microbiological training for laboratory staff and DSOs. Training modules included appropriate sample collection by DSOs, on-site laboratory training in detection of bacteria, and laboratory quality management system training.

Strengthening the capacity of public health systems to conduct surveillance, detect, and respond to infectious microbiological agents is essential in achieving the objectives of the global health security agenda. Through support of the US CDC, the Sekondi Public Health Laboratory was equipped to enhance their capacity to identify Enterobacteriaceae and non-Enterobacteriaceae from various samples including stool, blood, wound, urine, and others. From the sample investigation, *V. parahaemolyticus*, *A. hydrophila*, and *P. shigelloides* were isolated from stool and rectal samples of eight patients from Fijai in the Sekondi-Takoradi Municipality of the Western Region of Ghana. Although these pathogens are of public health importance, only a few reported cases are documented in Africa [6], with no reports from Ghana.

There are several bacterial etiological agents responsible for diarrhea outbreaks in developing countries. Typical among them, which cause moderate-to-severe cases include *Escherichia coli*, *Salmonella* (especially nontyphoidal *Salmonella*), *Campylobacter*, and *Shigella* species [7]. However, the pathogens identified in this study did not include any of these. *A. hydrophila* and *P. shigelloides* are Gram-negative bacilli within the families Aeromonadaceae and Enterobacteriaceae, respectively. *V. parahaemolyticus* shares family with *V. cholerae* in the group Vibrionaceae. Although the three bacteria occupy separate taxonomic niches, they are all widely distributed in brackish water, marine environments, drinking water, fresh water, and polluted waters [8]. These bacteria are associated with gastroenteritis accompanied by vomiting, and have been implicated in acute secretory, dysenteric, or choleric diarrhea [9]. Water and food serve as vehicles of transmission for *Aeromonas* and *Plesiomonas* organisms [10]. Transmission of *V. parahaemolyticus* is mainly through the consumption of contaminated seafood, especially oysters, causing acute gastroenteritis [11]. All three bacteria have been associated with diarrhea outbreaks.

The patients indicated they had eaten *kenkey* and *waakye* (local food made from maize and rice, respectively) prior to onset of the disease. It was not possible to confer a direct link to the food eaten as the source of infection since these patients ate these foods the night prior to the outbreak, and there was no possible way of collecting food samples for microbial screening. However, the community is situated alongside the sea and well noted for trading in seafoods including oysters, fish, and other crustaceans in its environs. Sea water, shell fish, and crustaceans are reservoirs of these bacteria, and possible risk factors for infection [12]. It is possible that consumption of the carbohydrate food and consumption of fish or some crustaceans are possible sources of infection in this outbreak. Virulence factors such as toxin-related hemolysin, capsular polysaccharide, lipases, enterotoxins, proteases, and other toxins are known to be responsible for pathogenesis of *V. parahaemolyticus*, *A. hydrophila*, and *P. shigelloides* [13, 14].

Our report is similar to outbreaks of diarrhea reported in other parts of Africa (Nigeria and Cameroon), Japan, and Bangladesh, which were all associated with *V. parahaemolyticus*, *P. shigelloides*, and *A. hydrophila* [15–18]. Klontz *et al.* similarly reported that patients infected with *A. hydrophila* or *P. shigelloides* displayed clinical features that were, mostly, similar to those infected with *V. cholerae* non-O1 [9].

In a typical endemic setting, especially in many communities in Africa, laboratory-based diagnostics are a challenge, and this results in the majority of diagnoses being made clinically and antimicrobials given empirically [19]. After confirmation of the various bacterial agents (by API) and subsequent ASTs, laboratory results were shared with the National Public Health and Reference Laboratory and the Disease Surveillance Unit as part of the IDSR system. This led to effective clinical management and discharge of the patients. Improper diagnosis usually leads to prolonged hospitalization, poor patient outcome, and likely increase in antimicrobial resistance. The current study emphasizes the need to build capacity for proper laboratory analysis for all diarrheal cases, which for a country like Ghana, could easily and erroneously be attributed to *V. cholerae*.

A major limitation to this study was the inability to confirm the identity of isolated pathogens with more accurate and sensitive molecular techniques such as polymerase chain reaction (PCR). This was primarily due to logistical and infrastructural challenges at the Sekondi Zonal PHL. Molecular techniques have been widely utilized in surveillance and other genetic studies of foodborne pathogens to increase understanding into the primary source of infection and genetic diversity.

One key lesson in this report is the resourcing and training of laboratory and DSOs, which enabled them to both detect and respond to this outbreak. All three identified bacteria cannot be detected easily with conventional microbiological techniques. The use of additional techniques, such as the API, enabled the Public Health Laboratory to detect these pathogens with a high degree of certainty. Resourcing and training of laboratory staff and DSOs in developing countries is essential in meeting the objectives of the Global Health Security Agenda.

## Conclusion

This report presents rare cases of diarrhea associated with *V. parahaemolyticus*, *A. hydrophila*, and *P. shigelloides*. These organisms might be widespread in Ghana, especially along the coastal towns, and may be significant causative agents of diarrheal outbreaks or might cause chronic diarrhea in immunocompromised patients. This report underscores the importance of laboratory analyses in outbreaks. Comprehensive investigation, including food microbiology for seafood, is recommended to map out sources of infection for better control measures.

## Data Availability

The dataset and laboratory protocols and/or materials used are available from the corresponding author on reasonable request.

## Abbreviations

APHL: Association of Public Health Laboratories
AST: Antimicrobial susceptibility test
CfHSS: Centre for Health System Strengthening
CLSI: Clinical and Laboratory Standards Institute
DSO: Disease surveillance officer
GHSA: Global Health Security Agenda
H_2_S: Hydrogen sulfide
IDSR: Integrated Disease Surveillance and Response
IHR: International Health Regulations
PHL: Public Health Laboratory
SARS: Severe acute respiratory syndrome
SIM: Sulfur indole motility
TCBS: Thiosulfate citrate bile salt sucrose
TSI: Triple sugar iron
US CDC: United States Centers for Disease Control and Prevention
WHO/AFRO: WHO Regional Office for Africa
WHO: World Health Organization
XLD: Xylose lysine deoxycholate
